# From empathy to creative output: exploring the emotional–cognitive mechanisms of digital creativity

**DOI:** 10.3389/fpsyg.2026.1749596

**Published:** 2026-02-25

**Authors:** Tianshu Li, Qizhe Zhao, Na Yang, Yaocheng Tian, Zhenwen Zheng, Zhixin Guo, Caisheng Liao

**Affiliations:** 1 School of Finance, City University of Macau, Macau, China; 2 School of Art and Design, Baise University, Baise, China; 3 Guangxi Zhuang Autonomous Region Museum, Nanning, China; 4 School of Management, Jinan University, Guangzhou, China; 5 League Committee, Nanning Normal University, Nanning, China; 6 Division of Electrical, Information and Communication Engineering, Kanazawa University, Kanazawa, Japan

**Keywords:** cognitive flexibility, digital creativity, digital empathy, digital self-efficacy, self-determination theory

## Abstract

The rapid advancement of digital technologies has reshaped how creativity is fostered, especially in fields such as education, business, and the creative industries. However, the mechanisms behind digital creativity remain underexplored, particularly regarding the roles of emotional and cognitive factors. Among these factors, empathy has long been considered a key driver of creativity; however, it remains unclear whether this mechanism still applies in digital environments. Guided by self-determination theory, this study proposes and tests an integrative model linking digital empathy, cognitive flexibility, digital self-efficacy, and digital creativity. Two complementary studies were conducted: a quasi-experimental study with university students and a survey with employees in digital workplaces. Structural equation modeling shows that digital empathy does not directly predict digital creativity but enhances it indirectly through cognitive flexibility. Digital self-efficacy strengthens the link between empathy and flexibility, amplifying this indirect effect. These results provide empirical evidence consistent with an emotional–cognitive pathway associated with digital creativity and suggest that empathy may function as a cognitively relevant factor rather than solely as an emotional response. By integrating emotion, cognition, and competence within a unified framework, this research extends existing creativity theory to digital contexts and offers practical insights for fostering innovation in educational and organizational settings.

## 1 Introduction

With the rapid advancement and widespread adoption of digital technologies, digital innovation has become a crucial driver of competitiveness and sustainable development across industries worldwide. Within this evolving context, digital creativity has emerged as a core capability that enables individuals to solve problems, generate new ideas, and foster innovation in digital environments (de Vasconcellos et al., 2021; Sun and Zhou, 2024). In particular, in fields such as education, the creative industries, and technological development, digital creativity directly determines the degree of innovation reflected in products and services. However, despite growing recognition among scholars and practitioners of its importance, cultivating digital creativity remains a complex challenge. This challenge extends beyond simply stimulating and maintaining individuals’ motivation for innovation, as it also involves designing effective support systems within increasingly dynamic and complex digital ecosystems (Samper-Márquez and Oropesa-Ruiz, 2025). Therefore, a deeper understanding of the mechanisms underlying digital creativity, especially the roles played by emotional and cognitive factors, is essential for promoting innovation in the digital era.

In recent years, research on digital creativity has made notable progress. Scholars have extensively discussed the factors influencing digital creativity, including technological factors (such as digital applications, systems, and educational media), individual internal factors (such as self-efficacy, motivation, and mindfulness), and external factors (such as teachers, learning systems, and organizational networks) (Tang et al., 2022; Wang and Shao, 2024; Wulan et al., 2025). However, as digital technologies continue to proliferate, new modes of work, communication, and collaboration are rapidly emerging. The widespread adoption of remote work and virtual collaboration, in particular, has reshaped how individuals and organizations operate (Chung et al., 2015; Bullini Orlandi et al., 2024). These transformations demand not only advanced technical skills and problem-solving abilities but also the capacity to build deep interpersonal connections in digital contexts (Laar et al., 2020). From a traditional perspective, empathy, defined as the ability to understand others’ emotions, attitudes, and intentions, and to engage in meaningful interpersonal interaction, is regarded as a key driver of creativity (Dostál et al., 2017; Tie et al., 2025). Yet it remains unclear whether this mechanism still holds in digital contexts. How, and to what extent, can digital empathy be translated into concrete creativity? To date, there has been a lack of systematic theoretical integration and empirical evidence addressing these questions. As a result, the transformation from digital empathy to digital creativity remains a theoretical black box that warrants deeper investigation.

To address the aforementioned research gaps, this study draws on self-determination theory (SDT) as an interpretative framework to examine how digital empathy may be associated with intrinsic motivation and digital creativity in ways that are conceptually consistent with SDT’s basic psychological needs (Deci and Ryan, 2000; Deci et al., 2017). Although creativity was not explicitly defined as a core outcome variable in the initial conception of SDT, recent research has widely applied SDT to explore how individuals can foster exploratory cognitive activities and creativity by satisfying basic psychological needs (Li et al., 2018; Fateh et al., 2023; Ahmed et al., 2024; Chen et al., 2025). According to SDT, intrinsic motivation is driven by the three basic psychological needs: relatedness, competence, and autonomy (Deci and Ryan, 2000). Within this theoretical perspective, digital empathy is conceptualized as a socio-emotional and relational experience that is relevant to the relatedness dimension of SDT, as it facilitates emotional connection and interpersonal understanding in digitally mediated interactions. Digital empathy provides a relational context that supports motivational engagement, through which emotional experiences may be internalized and translated into deeper cognitive involvement and digital creativity (Walther et al., 2017; De Dreu et al., 2024; Tie et al., 2025). This mechanism suggests that digital empathy can be understood as a relational experience relevant to the relatedness dimension of SDT, which supports motivational engagement in exploration and innovation and is associated with higher levels of digital creativity.

However, emotional activation alone may be insufficient to produce complex creativity directly. According to SDT, emotional experiences can satisfy the relatedness need and activate intrinsic motivation, but they generally need to be internalized through cognitive processes to contribute to creativity (Li et al., 2018; Ma et al., 2025). Specifically, cognitive flexibility, as an autonomy-oriented cognitive regulation mechanism, enables individuals to adjust their thinking strategies flexibly, integrating multiple perspectives, rules, and frameworks (Martin and Rubin, 1995). This cognitive flexibility allows emotional experiences to be reorganized and internalized, transforming them into innovative outputs (Peng et al., 2013; Li et al., 2018). In this process, cognitive flexibility functions as an autonomy-relevant cognitive regulation process, helping individuals maintain a sense of control over tasks when facing challenges, while also fostering the generation of creative ideas through cognitive restructuring. Therefore, cognitive flexibility plays a crucial mediating role between emotional experiences and creativity, transforming emotional motivation into cognitive activity and driving digital creativity (Lin et al., 2014; Tie et al., 2025).

Additionally, individuals’ belief in their digital capabilities, known as digital self-efficacy, plays a crucial role in this process (Hammer et al., 2021; Ulfert-Blank and Schmidt, 2022). From the SDT perspective, digital self-efficacy functions as a competence-relevant belief that enhances cognitive engagement in digital tasks (Deci and Ryan, 2000; Zhai and Li, 2025). Specifically, when individuals perceive themselves as having high levels of digital competence, they are more likely to sustain cognitive engagement when confronting complex digital tasks and convert empathy-driven understanding into innovative ideas, prototype development, or technological implementation. In contrast, lower digital self-efficacy may induce self-doubt or anxiety, weakening the transformation of emotional experience into creative performance. Therefore, the satisfaction of competence enhances individuals’ confidence, enabling them to maintain cognitive regulation in complex tasks and effectively translate emotional motivation into creativity (Yoo and Jang, 2023; Jeong and Jeong, 2025).

Building on the preceding analyses, this study articulates a process-oriented conceptual model of digital creativity that highlights the roles of empathic experience, cognitive regulation, and perceived digital competence. Without treating the focal variables as direct indicators of basic psychological needs, the findings are interpreted through a SDT–informed perspective to clarify how empathy-related experiences may be associated with creative outcomes *via* cognitive flexibility under varying levels of digital self-efficacy. By demonstrating the consistency of this pattern across student and workplace samples, the study provides convergent empirical support for a psychologically grounded account of digital creativity and offers practical insights for fostering innovative engagement in both educational and organizational contexts.

## 2 Review and hypothesis

### 2.1 Digital creativity

Digital creativity refers to an individual’s ability to identify problems, generate ideas, and express innovations by using digital tools and resources within technology-mediated environments. It represents an extension and reconfiguration of traditional creativity in the digital context (Lee and Chen, 2015; van Janse Rensburg et al., 2022). Compared with traditional creativity, digital creativity places greater emphasis on the integration of technological literacy, cognitive flexibility, and cross-domain collaboration, reflecting the evolution of human creative activity in an intelligent and interconnected era (Samper-Márquez and Oropesa-Ruiz, 2025). Early creativity research primarily focused on the fields of psychology and cognitive science, where scholars concentrated on the cognitive processes of individuals and methods for measuring creativity. Such as the initial studies of divergent thinking (Guilford, 1950) and creative personality traits (Torrance, 1966), which laid the foundation for later research on digital creativity. As psychological research progressed, scholars began to recognize that creativity is not only an individual cognitive process but also influenced by external environments and motivation. Amabile’s componential theory posits that creativity is the result of the interaction of three core components: domain-relevant skills, creativity-relevant cognitive processes (such as openness to ambiguity and risk-taking), and intrinsic motivation (Amabile, 1982; Amabile, 1983).

With the rapid development of information technology in the 21st century, unprecedented spaces for innovation have emerged, and digital technology has gradually become a key tool for driving creativity. Against this backdrop, the concept of digital creativity was proposed (Samper-Márquez and Oropesa-Ruiz, 2025; Wulan et al., 2025). Many scholars argue that digital creativity is driven by technology, focusing particularly on how technological tools directly facilitate the generation and expression of ideas, such as educational games and social platforms (Tang et al., 2022; Wulan et al., 2025). For example, the research by Chung et al. (2015) pointed out that the use of digital technology has a positive impact on both employees’ job performance and creativity. However, over time, some scholars have argued that improper use of technology can also hurt digital creativity (Wang et al., 2024). Consequently, scholars’ perspectives have gradually shifted from focusing solely on the use of technological tools to exploring factors such as emotional drive and cognitive adaptability, particularly how these internal factors work together to promote creativity (Bullini Orlandi et al., 2024; Wulan et al., 2025). This shift indicates that the generation of digital creativity no longer relies solely on technology itself; individual motivation, emotions, and psychological states are increasingly recognized as crucial factors.

At this stage, scholars have started to emphasize a “human-driven” perspective, arguing that internal factors such as emotions, psychological states, and motivation play a central role in the formation of digital creativity. For instance, some studies have pointed out that factors such as mindful learning and self-efficacy are believed to significantly enhance individuals’ creative performance (Yeh et al., 2019; Xu et al., 2025). However, this discussion has yet to explore in depth the interaction between technology and individual psychological states, particularly the lack of systematic research and empirical support regarding how emotional mechanisms and cognitive processes are transformed into innovative outcomes in digital environments. “Digital empathy,” as an interaction between technology and individual psychological states, provides a new perspective for understanding this issue. This interaction can stimulate emotional motivation and have a positive impact on cognitive flexibility, thereby driving the burst of innovative thinking (Lin et al., 2014; Tie et al., 2025). Based on this, this study attempts to explore how digital empathy, through stimulating emotional motivation and promoting cognitive flexibility, ultimately drives the generation of digital creativity by examining individual psychological mechanisms in combination with SDT.

### 2.2 Digital creativity: a SDT perspective

SDT posits that human behavior is shaped by the satisfaction of basic psychological needs that activate intrinsic motivation and guide behavior through internal regulatory processes (Deci and Ryan, 2000; Deci et al., 2017). This perspective is particularly relevant in digital contexts, where interactions are often characterized by reduced social cues, heightened uncertainty, and increased demands for autonomous decision-making. As a result, creative performance in digital environments depends not only on emotional engagement but also on how motivational activation is regulated and translated into creative action. From an SDT perspective, empathic engagement in digital interactions can function as an important motivational source for creativity by providing a relationally meaningful socio-emotional experience that is conceptually aligned with the relatedness dimension of self-determination theory and supports intrinsic motivation. At the same time, SDT suggests that the translation of such motivational activation into creative outcomes is not uniform across situations. In technologically complex and uncertain digital environments, emotionally driven motivation may, but does not necessarily, lead directly to creative performance. Instead, the extent to which empathy facilitates creativity often depends on whether motivational energy is internalized through self-regulated cognitive processes (Peng et al., 2013; Li et al., 2018).

Within this process, cognitive flexibility represents a key autonomy-related mechanism that enables individuals to explore multiple perspectives, integrate diverse information, and adapt cognitive strategies when engaging in digital creative tasks (Martin and Rubin, 1995; Dreu et al., 2011). In addition, digital self-efficacy reflects individuals’ perceived competence in digital environments and shapes their willingness to sustain autonomous cognitive engagement (Bandura, 1982; Moreira-Fontán et al., 2019). Thus, SDT provides a process-oriented framework in which emotional engagement, cognitive regulation, and competence beliefs jointly influence whether and how digital empathy is translated into digital creativity, while leaving open the possibility of both direct and indirect pathways.

### 2.3 Digital empathy and digital creativity

Empathy refers to the ability of individuals to understand others’ emotions, perspectives, and situations, and to respond appropriately based on that understanding. It is one of the core psychological mechanisms underlying social interaction and interpersonal relationships (Decety and Jackson, 2004). Empathy generally comprises two dimensions: affective empathy and cognitive empathy. The former involves emotional resonance and affective contagion, while the latter emphasizes perspective-taking, in which individuals adopt others’ viewpoints and mentally simulate their situations and thought processes (Decety and Jackson, 2004). As social interaction increasingly occurs in digital contexts, digital empathy represents an extension of traditional empathy into virtual forms of communication and collaboration (Terry and Cain, 2016; Collins et al., 2024). In such settings, digital empathy not only encompasses the perception and resonance of others’ emotions but also highlights individuals’ ability to reconstruct social connectedness and shared meanings through technological mediation (Collins et al., 2024).

This study focuses on cognitive digital empathy, which emphasizes individuals’ ability to understand others’ experiences through reasoning and perspective-taking in digital environments. Such empathic understanding allows individuals to experience emotional feedback associated with being understood and understanding others during virtual interactions, providing a relationally meaningful socio-emotional experience that is conceptually aligned with the relatedness dimension of self-determination theory, and is associated with enhanced feelings of belonging and psychological safety (Aharony and Rothschild, 2016; Deci et al., 2017). According to SDT, the satisfaction of relatedness needs plays a critical role in activating intrinsic motivation, which encourages individuals to engage more deeply in exploration-oriented cognitive activities rather than merely task-completion or compliance-driven behavior (Olafsen et al., 2025). In digital interactions, cognitive digital empathy enables individuals to reconsider problems from others’ perspectives, facilitating perspective integration and flexible meaning construction. It provides social and emotional resources that energize creative thinking, promote information integration and recombination, and enhance performance in divergent thinking tasks (Anderson et al., 2025). This intrinsically motivated cognitive engagement supports more inclusive and socially oriented innovative thinking (Zulaikha et al., 2023; Tie et al., 2025). Previous studies have also found that cognitive empathy is positively associated with both everyday creativity and domain-specific creative behavior (Tie et al., 2025). Furthermore, participants who exhibit empathy toward their targets tend to perform better on divergent thinking tasks, suggesting that empathy can stimulate creativity (Anderson et al., 2025). Accordingly, this study proposes the following hypothesis:

*H1*: Digital empathy is positively correlated with digital creativity.

### 2.4 Cognitive flexibility as a mediator

Cognitive flexibility refers to an individual’s ability to actively adjust thinking strategies and flexibly shift between different perspectives and conceptual frameworks when dealing with complex information or changing tasks (Martin and Rubin, 1995). When individuals exhibit higher levels of empathy in digital environments, they tend to be not only more emotionally sensitive but also more cognitively capable of perspective-taking and psychological role-shifting (Hargreaves et al., 2018). This process requires individuals to transcend egocentric thinking constraints and achieve cognitive reconciliation between the self and others, thereby enhancing the fluidity and openness of thought. SDT suggests that emotionally supportive contexts that are relevant to the relatedness dimension of self-determination theory promote autonomous motivation, which in turn encourages exploratory information processing and flexible switching among alternative interpretations (Deci et al., 2017; Olafsen et al., 2025). Digital empathy plays a crucial role as a form of cognitive activation, providing emotional support and a sense of social belonging that encourages individuals to explore new interpretative pathways and to switch more freely among different types of information (Deci et al., 2017; Tie et al., 2025). Empirical research has shown that learners with higher levels of digital empathy are more adept at perspective-taking and multi-angle communication in online learning and collaborative settings (Theophilou et al., 2024). Moreover, studies on teacher professionalization have empirically confirmed that empathic tendencies positively predict cognitive flexibility (Kaçay et al., 2021). These findings suggest that digital empathy, as a positive emotional experience, can enhance individuals’ cognitive flexibility by promoting multi-perspective thinking processes (Lin et al., 2014). Therefore, this study proposes the following hypothesis:

*H2a*: Digital empathy is positively correlated with cognitive flexibility.

Cognitive flexibility represents a core cognitive mechanism underlying creative thinking and functions as an autonomy-relevant cognitive regulation process that enables individuals to explore, integrate, and shift among alternative perspectives (Dreu et al., 2011; Kılıç et al., 2024). When individuals feel free to explore, experiment, and make independent decisions, they tend to demonstrate greater cognitive openness and integrative capacity (Li et al., 2018; Olafsen et al., 2025; Ren and Zhang, 2025). According to SDT, satisfying the need for autonomy significantly enhances individuals’ exploratory tendencies and their ability to flexibly switch between cognitive frameworks during information processing, thereby fostering higher levels of creativity in complex tasks (Deci et al., 2017; Li et al., 2018; Ren and Zhang, 2025). Moreover, some empirical studies have shown that individuals with high cognitive flexibility are more capable of breaking away from habitual mental sets, reorganizing information structures, and generating novel and valuable ideas (Dreu et al., 2011; Shao et al., 2018). In digital contexts, this ability becomes particularly critical, as an open cognitive state enables individuals to transcend media constraints and semantic boundaries, transforming diverse sources of information into innovative forms of expression. Therefore, this study proposes the following hypothesis:

*H2b*: Cognitive flexibility is positively correlated with digital creativity.

In summary, digital empathy stimulates individuals’ intrinsic motivation through emotional connection, creating a sense of psychological safety and social belonging that enables them to reconstruct problems from others’ perspectives and to engage in flexible thinking. Building on this foundation, cognitive flexibility further promotes autonomous exploration and innovative expression by transforming emotional energy into creative cognitive processing. In other words, digital empathy can be understood as a relationally meaningful socio-emotional experience that is conceptually aligned with the relatedness dimension of self-determination theory and may activate intrinsic emotional engagement, while cognitive flexibility represents an autonomy-relevant cognitive regulation process that supports the internalization of this engagement and facilitates its translation into digital creativity (Lin et al., 2014; Deci et al., 2017). Therefore, this study proposes the following hypothesis:

*H2c*: Cognitive flexibility plays a mediating role in the relationship between digital empathy and digital creativity.

### 2.5 Digital self-efficacy as a moderator

Digital self-efficacy refers to individuals’ belief in their ability to effectively use digital tools to complete tasks and solve problems, and functions as a competence-relevant belief that shapes individuals’ confidence and engagement in digital environments (Deci and Ryan, 2000; Hammer et al., 2021; Ulfert-Blank and Schmidt, 2022). SDT posits that when individuals perceive themselves as capable and effective in interacting with their environment, they are more likely to experience autonomous motivation, which facilitates the translation of emotional engagement into purposeful and sustained action (Deci et al., 2017; Olafsen et al., 2025). Individuals with strong self-efficacy are more likely to engage proactively, persist in their efforts, and experience greater agility and creative satisfaction (Maran et al., 2022; Yang and Zhou, 2022). Conversely, those with low self-efficacy tend to experience anxiety in the face of technological uncertainty, which weakens the intrinsic motivation elicited by empathy (Deng and Liu, 2025). Existing research further suggests that digital self-efficacy enhances creativity by increasing individuals’ sense of control and confidence in digital environments. For example, Voigt et al. (2019) demonstrated that self-efficacy in design thinking activities strengthens the transformation of empathy into creative action. Similarly, the study by Jeong and Jeong (2025) indicates that individuals with high creative self-efficacy are more likely to view difficult creative tasks as opportunities for growth rather than as threats, thereby adopting more adaptive and creative problem-solving approaches. These findings indicate that digital self-efficacy not only enhances individuals’ confidence in digital contexts but also amplifies the effect of empathy on creativity. Therefore, this study proposes the following hypothesis:

*H3a*: Digital self-efficacy positively moderates the relationship between digital empathy and digital creativity, such that the higher the digital self-efficacy, the stronger the relationship between digital empathy and digital creativity.

Additionally, an increase in digital self-efficacy can enhance the transformation process from empathy to cognitive flexibility. SDT argued that the fulfillment of the need for competence enhances individuals’ sustained motivation and psychological energy, enabling them to handle complex tasks with greater flexibility (Deci et al., 2017). Ren et al. (2025) point out that employees with high levels of digital self-efficacy are more likely to approach digital challenges with a problem-solving mindset, draw on multiple information sources, and integrate new knowledge into creative work processes. Similarly, Hu et al. (2023) found that internet users with higher self-efficacy exhibited stronger cognitive regulation and perspective-taking abilities when responding to social stimuli. Based on these findings, it can be inferred that when individuals in digital environments experience both emotionally supportive interpersonal connections that are relevant to the relatedness dimension of self-determination theory and a strong sense of capability grounded in competence-relevant beliefs, the emotional energy triggered by digital empathy is more likely to be transformed into flexible thinking and innovative expression. In other words, digital self-efficacy not only strengthens the effect of digital empathy on cognitive flexibility but also amplifies the indirect pathway through which this relationship promotes creativity. Therefore, this study proposes the hypotheses:

*H3b*: Digital self-efficacy positively moderates the relationship between digital empathy and cognitive flexibility, such that the higher the digital self-efficacy, the stronger the relationship between digital empathy and cognitive flexibility.

*H3c*: Digital self-efficacy moderates the indirect relationship between digital empathy and digital creativity through cognitive flexibility, such that this indirect relationship becomes stronger at higher levels of digital self-efficacy.

**Figure 1:**
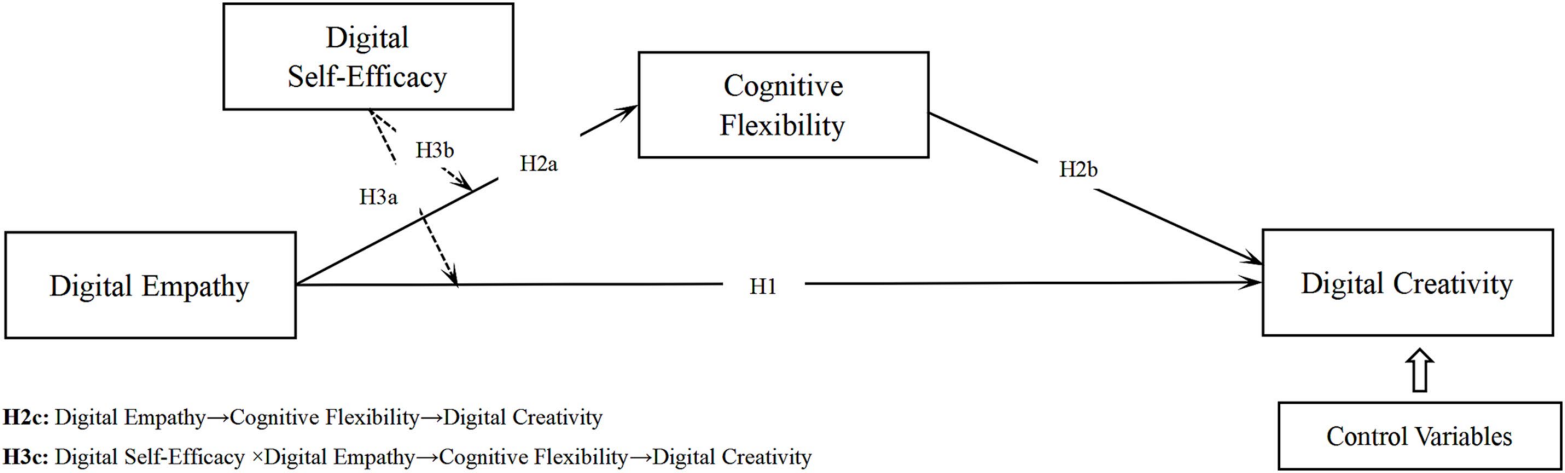
The conceptual model of this study.

This study proposes a conceptual model examining how digital empathy influences digital creativity. Drawing on an SDT-informed perspective, the model illustrates how digital empathy, cognitive flexibility, and digital self-efficacy interact across emotional, cognitive, and competence-related levels (see Figure 1).

## 3 Methodology

### 3.1 Research design

This study adopted a two-stage design to balance internal control and ecological validity. Study 1 employed a scenario-based quasi-experimental design to examine the psychological mechanisms linking digital empathy, cognitive flexibility, and digital creativity among university students majoring in design, computer science, and creative media. In Study 1, participants completed a digital empathy activation task based on a simulated online brand–consumer interaction scenario, which was designed to induce high versus low levels of digital empathy. Rather than evaluating the objective quality of creative outputs, the design focused on activating empathic cognitive–emotional states and examining their effects on subsequent psychological variables, including cognitive flexibility and self-perceived digital creativity. Following the empathy activation task, participants completed validated self-report measures of digital empathy, cognitive flexibility, digital self-efficacy, and digital creativity. Study 2 surveyed employees from creative, technological, and business sectors to examine the generalizability of the proposed mechanism and the moderating role of digital self-efficacy in real-world contexts. Data from both studies were analyzed using partial least squares structural equation modeling (PLS-SEM), which is suitable for mechanism-oriented and predictive research (Hair et al., 2019).

### 3.2 Measures

This study adopted validated and localized measurement scales, all rated on a seven-point Likert scale. Four core constructs were assessed: digital empathy, cognitive flexibility, digital self-efficacy, and digital creativity. Digital empathy was measured using six items adapted from the digital perspective-taking dimension of Collins et al. (2024). Cognitive flexibility was measured with twelve items revised from Martin and Rubin (1995) to fit digital task contexts. Digital self-efficacy included seven items adapted from Hammer et al. (2021), and digital creativity was assessed with three items adapted from Shao et al. (2022). All scales underwent bilingual translation, back-translation, and pilot testing to ensure semantic equivalence and reliability. The full list of items and sources is provided in Table 1.

**Table 1:** Scales used in study 1 and study 2.

Construct and item	Loading (study 1)	Loading (study 2)
Cognitive flexibility (study 1: Cronbach’s *α* = 0.960, CR = 0.965, AVE = 0.694; study 2: Cronbach’s *α* = 0.958, CR = 0.963, AVE = 0.684)		
CF1. I can express an idea in various digital formats (e.g., text, image, video).	0.841	0.839
CF2. I avoid exploring new or unfamiliar digital tools. (R)	0.858	0.857
CF3. I feel that I rarely have the chance to make independent decisions in creative tasks. (R)	0.839	0.825
CF4. I can find effective digital solutions to seemingly unsolvable problems.	0.820	0.812
CF5. I seldom have choices when deciding how to approach a digital project. (R)	0.854	0.861
CF6. I am willing to experiment with creative methods to solve problems.	0.841	0.837
CF7. I can adapt my behavior appropriately in different digital situations.	0.839	0.812
CF8. My actions are based on conscious and deliberate decisions.	0.817	0.811
CF9. I can find multiple ways to deal with a digital challenge.	0.831	0.826
CF10. I find it difficult to apply my knowledge in real digital contexts. (R)	0.834	0.833
CF11. I am open to listening to and considering alternative ways of solving problems.	0.831	0.840
CF12. I have the confidence to try different approaches when working digitally.	0.788	0.767
Digital creativity (study 1: Cronbach’s α = 0.857, CR = 0.913, AVE = 0.777; study2: Cronbach’s α = 0.852, CR = 0.910, AVE = 0.771)		
DC1. I suggest new and creative ways to improve study or work performance through using digital tools.	0.889	0.877
DC2. I come up with innovative solutions to problems by using digital technologies.	0.877	0.875
DC3. I often generate new and original ideas when engaging in digital design or creative work.	0.879	0.883
Digital empathy (study1: Cronbach’s α = 0.943, CR = 0.954, AVE = 0.777; study2: Cronbach’s α = 0.923, CR = 0.940, AVE = 0.722)		
DE1. When communicating online, I try to understand others’ emotions and perspectives.	0.909	0.844
DE2. Before replying to someone’s message, I imagine how they might feel.	0.883	0.860
DE3. During online discussions, I consider what others might be thinking or experiencing.	0.886	0.857
DE4. I pay attention to how my online comments might affect others’ feelings.	0.867	0.888
DE5. I can easily put myself in others’ shoes during digital interactions.	0.900	0.848
DE6. When people disagree online, I try to understand both sides of the argument.	0.844	0.797
Digital self-efficacy (study 1: Cronbach’s α = 0.947, CR = 0.952, AVE = 0.740; study 2: Cronbach’s α = 0.957, CR = 0.963, AVE = 0.79)		
DS1. I am confident in my ability to use various digital devices and platforms.	0.903	0.857
DS2. I can quickly learn how to use unfamiliar digital tools.	0.867	0.860
DS3. I can give advice to others when they want to choose or use digital tools.	0.855	0.917
DS4. I feel comfortable completing creative or design tasks that require digital skills.	0.910	0.917
DS5. I can usually solve problems that occur with digital devices.	0.822	0.900
DS6. I can help others when they have difficulties using digital devices.	0.842	0.894
DS7. When technology malfunctions, I feel helpless and frustrated. (R)	0.818	0.872

## 4 Study 1

### 4.1 Participants and procedure

Participants in Study 1 were undergraduate students enrolled in majors related to digital creativity, including design, computer science, and creative media. A total of 240 students were recruited to take part in the study, and 197 valid responses were retained after excluding incomplete questionnaires, highly repetitive responses, or failed attention checks. Among the valid participants, 45.7% were male, with an average age of 20.350 years (SD = 1.65). Data collection was conducted in offline laboratory settings between April and May 2025.

Study 1 employed a scenario-based quasi-experimental design with a single factor (digital empathy activation: high *vs.* low). The task materials consisted of a simulated online comment scenario depicting interactions between brand designers and consumers. Participants’ levels of digital empathy were activated through a reading and response task rather than through the evaluation of creative outputs. In the high-empathy condition, participants were instructed to imagine themselves as the brand designer and to actively understand consumers’ emotions, expectations, and underlying values. In the low-empathy condition, participants were asked to adopt the perspective of an external observer and to describe the content of the comments in an objective and emotionally neutral manner, without interpreting consumers’ feelings or intentions. Following the empathy activation task, all participants completed self-report questionnaires measuring digital empathy, cognitive flexibility, digital self-efficacy, and perceived digital creativity. The entire procedure took approximately 10–20 min to complete.

### 4.2 Data quality assessment

To ensure the robustness and reliability of the data for subsequent analyses, we first examined the distributional properties of the study variables. The results of the normality analysis indicated that the skewness and kurtosis values of all variables fell within the acceptable range of ±2, satisfying the assumption of normal distribution (Vaithilingam et al., 2024; Liao et al., 2025). Given that all variables were collected using self-reported measures, we further assessed the potential risk of common method bias (CMB). Specifically, two complementary approaches were employed. Harman’s single-factor test indicated that the first factor accounted for 39.671% of the total variance, below the 50% threshold, suggesting low CMB risk (Podsakoff et al., 2003). Moreover, a common method factor model was also tested, revealing average standardized loadings of 0.858 (*p* < 0.001) on substantive factors and 0.000 on the method factor, with most method loadings nonsignificant. The variance ratio of approximately 46:1 indicated that the method factor’s effect was negligible (Liang et al., 2007), confirming that no serious CMB existed in the data.

### 4.3 Manipulation check

To verify the effectiveness of the empathy manipulation, an independent samples t-test was conducted using participants’ scores on the digital empathy scale as the dependent variable. The results showed that the high-empathy group (M = 5.42, SD = 0.59) scored significantly higher than the low-empathy group (M = 2.24, SD = 0.58), and the difference was statistically significant, *t* (195) = 38.27, *p* < 0.001, 95% CI [3.01, 3.34]. These findings indicate that the manipulation was successful, as participants in the high-empathy condition exhibited significantly higher levels of digital empathy than those in the low-empathy condition, indicating that the scenario task effectively activated distinct digital empathy states.

### 4.4 Evaluating the measurement model

In this study, the Cronbach’s *α* values for all constructs exceeded 0.7, and the composite reliability (CR) ranged from 0.913 to 0.965, indicating high internal consistency of the scales (Hair et al., 2019). The average variance extracted (AVE) values were all greater than 0.5, suggesting good convergent validity for each construct. Moreover, as shown in Table 2, the square roots of the AVE values for all latent variables were higher than their correlations with other constructs, and the HTMT ratios were all below 0.85, further confirming satisfactory discriminant validity among the constructs (Henseler et al., 2015).

**Table 2:** Results of model discriminant validity.

Constructs	Fornell-larcker criterion					HTMT ratio			
	1	2	3	4		1	2	3	4
1. CF	**0.833**								
2. DC	0.566	**0.882**				0.623			
3. DE	0.529	0.351	**0.882**			0.547	0.385		
4. DS	0.103	0.049	−0.055	**0.860**		0.090	0.065	0.087	

The bold elements are the square roots of AVE, CF, Cognitive flexibility; DC, Digital creativity; DE, Digital empathy; DS, Digital self-efficacy.

### 4.5 Hypothesis testing

After establishing the reliability and validity of the measurement model, the structural model was evaluated using SmartPLS 4.0 with a bootstrapping procedure of 5,000 resamples. Before examining the hypothesized relationships, the overall quality of the structural model was first assessed. The results indicate that the model exhibits adequate explanatory power, with *R^2^* values ranging from 0.280 to 0.419 across the endogenous constructs. In addition, all *Q^2^* values are greater than zero, demonstrating satisfactory predictive relevance (Hair et al., 2019). The variance inflation factor (VIF) values range from 1.000 to 1.399, suggesting that multicollinearity does not pose a concern for the estimation of the structural relationships (Hair et al., 2019).

With the structural model quality confirmed, the analysis then proceeded to estimate the hypothesized effects. First, the direct and indirect effects were examined based on the baseline model, while controlling for participants’ gender, age, and familiarity with digital tools. The hypothesis testing results are summarized in Table 3. The direct relationship between digital empathy and digital creativity is not statistically significant (*β* = 0.072, *p* = 0.364), and therefore H1 is not supported. However, digital empathy is positively associated with cognitive flexibility (*β* = 0.529, *p* < 0.001), and cognitive flexibility, in turn, positively predicts digital creativity (*β* = 0.529, *p* < 0.001), supporting H2a and H2b. Consistent with this pattern, the indirect effect of digital empathy on digital creativity *via* cognitive flexibility is significant (*β* = 0.280, *p* < 0.001), providing support for H2c.

**Table 3:** Results of hypothesis testing.

Path	*β*-value	LLCI	ULCI	*p*-value	*R^2^*	*Q^2^*	*VIF*	Support
Direct and indirect effect results								
H1: DE → DC	0.072	−0.083	0.226	0.364			1.390	No
H2a: DE → CF	0.529	0.423	0.636	0.000	0.280	0.191	1.000	Yes
H2b: CF → DC	0.529	0.38	0.666	0.000			1.399	Yes
H2c: DE → CF → DC	0.280	0.191	0.381	0.000	0.328	0.241		Yes
Moderating effect results								
H3a: DS *x* DE → DC	−0.092	−0.245	0.088	0.279			1.261	No
H3b: DS *x* DE → CF	0.355	0.122	0.450	0.000	0.419	0.286	1.001	Yes
H3c: DS *x* DE → CF → DC	0.204	0.066	0.286	0.000	0.335	0.242		Yes

CF, Cognitive flexibility; DC, Digital creativity; DE, Digital empathy; DS, Digital self-efficacy; LLCI, Lower limit confidence interval; ULCI, Upper limit confidence interval.

After establishing the main and mediating effects in the baseline model, the analysis further examined whether these relationships are contingent upon individual differences in digital self-efficacy. Accordingly, moderating and moderated mediation effects were tested using the interaction modeling procedure implemented in SmartPLS 4.0, in which interaction constructs were specified directly within the structural model. The results indicate that digital self-efficacy does not significantly moderate the direct relationship between digital empathy and digital creativity (*β* = −0.092, *p* = 0.279), and thus H3a is not supported. In contrast, digital self-efficacy significantly moderates the relationship between digital empathy and cognitive flexibility (*β* = 0.355, *p* < 0.001), indicating that the positive effect of digital empathy on cognitive flexibility becomes stronger at higher levels of digital self-efficacy, supporting H3b. Given that the moderating effect occurs on the first stage of the indirect pathway, the final step of the analysis examined whether this conditional effect extends to the overall indirect relationship. The results show that digital self-efficacy significantly strengthens the indirect effect of digital empathy on digital creativity through cognitive flexibility (*β* = 0.204, *p* < 0.001), thereby supporting H3c.

## 5 Study 2

### 5.1 Participants and procedure

Study 2 targeted a workplace sample to examine the applicability and robustness of the proposed model in real organizational contexts. Data were collected online between June and July 2025 using a combination of non-probability snowball sampling and industry-based stratified control to ensure representation across major sectors, including creative industries, information technology, and business operations. To minimize CMB, several procedural controls were applied, such as emphasizing anonymity and confidentiality, randomizing item order across constructs, and including reverse-coded items. A total of 386 questionnaires were collected, and after removing incomplete, inconsistent, or failed attention-check responses, 303 valid samples were retained (45.9% male; M = 27.35 years, SD = 7.05).

### 5.2 Data quality assessment

In this study, the skewness and kurtosis values of all variables were within the range of ±2, indicating that the data distribution met the assumption of normality (Vaithilingam et al., 2024; Liao et al., 2025). Harman’s single-factor test showed that the first factor accounted for 37.600% of the total variance, below the 50% threshold (Podsakoff et al., 2003). In addition, the average loading on substantive factors was 0.853 (*p* < 0.001), while that on the method factor was 0.000 and mostly nonsignificant, with a variance ratio of approximately 61:1. These results suggest that there was no significant common method bias, and the data were suitable for subsequent analyses (Liang et al., 2007).

### 5.3 Evaluating the measurement model

For the measurement model, all constructs demonstrated satisfactory reliability, with Cronbach’s *α* values exceeding 0.7 and CR values ranging from 0.910 to 0.963, indicating high internal consistency. The AVE values were all above 0.5, confirming good convergent validity. Regarding discriminant validity, as shown in Table 4, the square roots of the AVE values for all latent variables were greater than their correlations with other variables, and the HTMT ratios were below 0.85, further supporting adequate discriminant validity among the constructs (Henseler et al., 2015).

**Table 4:** Results of model discriminant validity.

Constructs	Fornell-larcker criterion					HTMT ratio			
	1	2	3	4		1	2	3	4
1. CF	**0.827**								
2. DC	0.481	**0.878**				0.530			
3. DE	0.518	0.275	**0.849**			0.546	0.309		
4. DS	0.048	0.100	−0.040	**0.888**		0.058	0.097	0.061	

The bold elements are the square roots of AVE, CF, Cognitive flexibility; DC, Digital creativity; DE, Digital empathy; DS, Digital self-efficacy.

### 5.4 Hypothesis testing

In this study, the structural model assessment indicates adequate explanatory power, with *R^2^* values ranging from 0.241 to 0.418 across the endogenous variables. All *Q^2^* values are greater than zero, suggesting satisfactory predictive relevance. The VIF values range from 1.000 to 1.381, indicating that multicollinearity is not a concern.

The analytical software and methods in Study 2 are consistent with those in Study 1. After controlling for age, gender, education level, and familiarity with digital tools, the hypothesis testing results are presented in Table 5. The relationship between digital empathy and digital creativity is not significant (*β* = 0.027, *p* = 0.666); thus, H1 is not supported. Digital empathy is positively associated with cognitive flexibility (*β* = 0.518, *p* < 0.001), and cognitive flexibility is positively related to digital creativity (*β* = 0.461, *p* < 0.001), supporting H2a and H2b. Furthermore, digital empathy demonstrates an indirect relationship with digital creativity through cognitive flexibility (*β* = 0.239, *p* < 0.001), supporting H2c. Regarding moderating effects, digital self-efficacy does not significantly moderate the relationship between digital empathy and digital creativity (*β* = 0.035, *p* = 0.571; H3a not supported). However, it significantly moderates the relationship between digital empathy and cognitive flexibility (*β* = 0.386, *p* < 0.001; H3b supported) and further strengthens the overall indirect relationship (*β* = 0.168, *p* < 0.001; H3c supported).

**Table 5:** Results of hypothesis testing.

Path	*β*-value	LLCI	ULCI	*p*-value	*R^2^*	*Q^2^*	*VIF*	Support
Direct and indirect effect results								
H1: DE → DC	0.027	−0.095	0.158	0.666			1.381	No
H2a: DE → CF	0.518	0.431	0.603	0.000	0.269	0.181	1.000	Yes
H2b: CF → DC	0.461	0.33	0.588	0.000			1.381	Yes
H2c: DE → CF → DC	0.239	0.167	0.322	0.000	0.241	0.171		Yes
Moderating effect results								
H3a: DS *x* DE → DC	0.035	−0.091	0.159	0.571			1.136	No
H3b: DS *x* DE → CF	0.386	0.258	0.466	0.000	0.418	0.282	1.012	Yes
H3c: DS *x* DE → CF → DC	0.168	0.090	0.242	0.000	0.248	0.174		Yes

CF, Cognitive flexibility; DC, Digital creativity; DE, Digital empathy; DS, Digital self-efficacy; LLCI, Lower limit confidence interval; ULCI, Upper limit confidence interval.

### 5.5 Measurement invariance of composite models (MICOM) analysis

Given that the present study integrates data from two distinct samples, namely students and employees, measurement invariance was examined before estimating the integrated structural model to ensure that the constructs were interpreted equivalently across groups. To this end, the MICOM procedure was employed to assess whether the measurement models met the requirements for sample integration, following established guidelines for variance-based structural equation modeling (Henseler et al., 2016; Shafaei et al., 2019).

As shown in Table 6, the MICOM results indicate that the measurement models satisfy the conditions required for meaningful integration of the student and employee samples. Although variance invariance was not supported for digital empathy, full measurement invariance was confirmed for cognitive flexibility, digital creativity, and digital self-efficacy, while digital empathy exhibited partial measurement invariance. These results provide sufficient evidence of measurement invariance to justify the integration of the two samples and the estimation of a unified structural model.

**Table 6:** Results of 3-step measurement invariance testing using permutation (student vs employee).

Constructs	Step 1	Step 2			Step 3a (mean)				Step 3b (variance)				Measurement invariance
	Configural invariance	C = 1	5.0% quantile of C_u_	Compositional invariance	Differences	2.5%	97.5%	Equal mean value	Differences	2.5%	97.5%	Equal variance	
CF	Yes	1.000	1.000	Yes	−0.163	−0.180	0.189	Yes	0.019	−0.146	0.145	Yes	Full
DC	Yes	1.000	0.999	Yes	−0.153	−0.185	0.176	Yes	−0.034	−0.198	0.175	Yes	Full
DE	Yes	1.000	0.999	Yes	−0.008	−0.175	0.182	Yes	0.448	−0.149	0.134	No	Partial
DS	Yes	0.987	0.651	Yes	−0.028	−0.173	0.178	Yes	−0.098	−0.131	0.134	Yes	Full

CF, Cognitive flexibility; DC, Digital creativity; DE, Digital empathy; DS, Digital self-efficacy.

## 6 Conclusions and general discussion

### 6.1 Main conclusions

This study explores the relationships among digital empathy, cognitive flexibility, and digital creativity through two complementary sub-studies and analyzes the moderating effect of digital self-efficacy. The results highlight the interactive pattern of emotional, cognitive, and competence-related beliefs associated with digital creativity, and provide empirical support for the core hypotheses tested across the two sub-studies. Firstly, the study found that the direct relationship between digital empathy and digital creativity was not significant. This result differs from patterns reported in prior emotion–creativity research, where empathy has often been found to be directly associated with creativity through emotional motivation (Alzayed et al., 2022; Li et al., 2024a; Tie et al., 2025). In this study, although digital empathy constitutes a relationally meaningful socio-emotional experience that is conceptually aligned with the relatedness dimension of self-determination theory and stimulates intrinsic emotional engagement, this engagement does not appear to directly translate into digital creativity (Deci and Ryan, 2000; Ryan et al., 2022). In this context, the stimulation of emotional motivation may only serve as the starting point of the innovation process, rather than being a direct driving force. As suggested by some studies, emotional motivation requires mediation through cognitive mechanisms to translate into actual creativity (Lin et al., 2014; Tie et al., 2025). That is, while emotional motivation stimulates cognitive activity, without the involvement of higher-order cognitive processing mechanisms such as cognitive flexibility, it may not directly foster the generation of creative ideas. Furthermore, digital empathy may be moderated by situational factors (Powell and Roberts, 2017; Čekić, 2025). In this study, although empathy was stimulated through situational simulations, the complexity of the actual digital environment could affect the effect of empathy on the translation into innovative behaviors. Therefore, the impact of digital empathy may vary in strength and direction across different contexts.

Further analyses indicated a statistically significant mediating role of cognitive flexibility in the relationship between digital empathy and digital creativity. From an SDT-informed perspective, this pattern can be interpreted as indicating that emotional engagement may be translated into innovative behavior through cognitive mechanisms (Dreu et al., 2011; Li et al., 2018, 2024b). Cognitive flexibility enables individuals to effectively adjust their thinking strategies and flexibly respond to changes in tasks, thereby generating new and innovative solutions. Individuals with higher digital empathy demonstrate greater cognitive flexibility, allowing them to better integrate information and view problems from multiple perspectives, which leads to more innovative ideas when facing digital tasks. Relatedly, the importance of cognitive flexibility in this study further confirms that cognitive flexibility is not merely a “tool” for innovation but a key bridge for transforming emotional motivation into creativity (Lin et al., 2014). Cognitive flexibility helps individuals break fixed thinking patterns, reorganize information, and fosters greater creativity in the face of complex tasks (Olafsen et al., 2025; Ren and Zhang, 2025). Therefore, although the direct relationship between digital empathy and digital creativity is not significant, digital empathy can still indirectly enhance creativity through the mediating role of cognitive flexibility.

Regarding digital self-efficacy, the results show that digital self-efficacy significantly moderates the relationship between digital empathy and cognitive flexibility. This finding suggests that an individual’s confidence in their digital abilities can enhance cognitive flexibility, which in turn promotes innovative performance. This is consistent with the study by Jeong and Jeong, (2025), which found that digital self-efficacy facilitates cognitive regulation, making individuals more flexible and innovative when faced with complex tasks. However, digital self-efficacy did not significantly moderate the direct relationship between digital empathy and digital creativity. This non-significant result may be related to the complexity and multidimensional nature of digital creativity. Digital creativity not only depends on the stimulation of emotional and cognitive factors but is also influenced by external environmental factors and task complexity. Therefore, although digital self-efficacy has a significant moderating effect on cognitive flexibility, more external support and contextual conditions may be required to achieve a more significant impact on the final expression of digital creativity.

### 6.2 Theoretical implications

This study is theoretically informed by SDT and examines how digital empathy, cognitive flexibility, and digital self-efficacy are jointly associated with digital creativity within digital environments. Although SDT originally did not focus on creativity and innovative behavior (Deci and Ryan, 2000; Deci et al., 2017), as research has progressed, the application of SDT has gradually expanded to explain the process of creativity generation (Fateh et al., 2023; Ahmed et al., 2024; Chen et al., 2025). Building on this stream of work, the present study proposes a multidimensional interactive model grounded in SDT to explain how emotional, cognitive, and competence-related factors jointly shape digital creativity. The results indicate that digital empathy is indirectly associated with digital creativity through cognitive flexibility, suggesting a mediating pathway linking emotional engagement to creative outcomes in digital contexts. Interpreted through an SDT framework, cognitive flexibility reflects autonomy-related cognitive regulation, whereas digital self-efficacy represents a competence-related condition that influences how this pathway operates. These findings advance current understanding of digital creativity by clarifying how emotional experience, cognitive regulation, and competence beliefs interact within a digitally mediated creative process.

Furthermore, this study provides empirical evidence consistent with the mediating role of cognitive flexibility in the association between emotional activation and creative outcomes, offering theoretically informed insights into how emotional experiences may be translated into creativity through cognitive regulation processes. Prior research has often examined relatedness needs (such as empathy) as factors associated with creativity (Li et al., 2024a; Tie et al., 2025), while paying comparatively less attention to the cognitive processes through which emotional experiences may be linked to creative outcomes. However, the findings indicate that digital empathy does not directly enhance digital creativity but is indirectly associated with creative outcomes through cognitive flexibility, which can be theoretically interpreted as a cognitive regulation process (Li et al., 2018). This finding adds a process-oriented nuance to existing emotion–creativity research by highlighting a cognitively mediated association through which emotional understanding may be linked to innovative behavior. Based on the observed pattern of results, emotional experiences (such as digital empathy) may be associated with innovative outcomes through cognitively mediated processes involving cognitive flexibility, highlighting the role of emotion–cognition interactions in creativity (Lin et al., 2014; Tie et al., 2025). These findings provide an SDT-consistent lens for interpreting the emotion–creativity relationship in digital contexts.

Finally, this study identifies a pathway-specific role of digital self-efficacy, indicating that self-efficacy may condition digital creative processes through its association with cognitive flexibility rather than uniformly moderating the direct link between digital empathy and creativity. Prior research has often examined self-efficacy as a factor directly associated with creativity (Islam and Asad, 2024; Jeong and Jeong, 2025), but this study finds that, although digital self-efficacy does not significantly enhance the direct impact of digital empathy on digital creativity, it indirectly promotes creative generation through its effect on cognitive flexibility. This pattern suggests that, in digital creative contexts, the influence of self-efficacy may be more closely reflected in cognitive regulation processes (Hu et al., 2023; Oh and Pyo, 2023) rather than in direct creative output. These findings provide a contextualized and process-oriented interpretation of how competence beliefs operate in digital creativity, highlighting the importance of cognitive adaptability as an intermediary mechanism.

### 6.3 Practical implications

The proposed model in this study offers a multi-level framework for fostering digital creativity within both educational and organizational contexts. In educational practice, curriculum design should place greater emphasis on guiding empathic experiences. Educators can introduce authentic problems, role-playing exercises, or cross-cultural tasks that allow learners to understand others’ perspectives within digital spaces, thereby cultivating a self-initiated motivation for exploration. Such contexts encourage students to shift their focus from merely “solving tasks” to “understanding people,” grounding creative thinking in emotional and cognitive engagement. Meanwhile, teaching activities should provide space for cognitive transformation. For example, during group collaboration or digital creation tasks, students can be encouraged to experiment with diverse modes of expression and problem-solving strategies, thereby enhancing their cognitive flexibility. Creativity does not stem from the accumulation of information but from the variability of thought. Finally, schools and teachers should attend to learners’ beliefs in their competence, offering tiered feedback, opportunities for presentation, and self-efficacy training.

In organizational management, innovation emerges from the joint shaping of structure and culture. Managers should regard empathy not merely as an interpersonal skill but as a core element of organizational communication. Allowing employees to feel understood and respected during project discussions and digital collaboration can significantly enhance psychological safety within teams. Psychological safety does not equate to leniency; rather, it serves as a foundation of trust that encourages individuals to express divergent ideas. At the same time, organizations should reduce excessive procedural control and leave room for employees to think and experiment. Providing spaces for experimentation and mechanisms for interim sharing can make innovative behaviors a natural part of organizational functioning rather than an additional burden. Moreover, through continuous training and experience-sharing, organizations can help employees recognize and strengthen their digital competence beliefs, enabling them to maintain confidence and agency amid change.

At the societal level, policymakers and educational institutions can draw on these insights to construct a more sustainable innovation ecosystem. The cultivation of digital literacy should transcend technical training to become a systematic project encompassing emotional understanding, cognitive openness, and competence development. Educational assessment, corporate training, and social incentive systems should jointly support the growth of these multidimensional abilities, extending the cultivation of creativity from individual experiences to broader cultural contexts. Through policy alignment and coordinated resource allocation, it is possible to foster a virtuous cycle between emotional understanding and innovative behavior on a societal scale.

### 6.4 Limitations and future directions

Despite its contributions, this study has several limitations that should be acknowledged. First, although this research was conceptually grounded in SDT, it did not directly assess the satisfaction of basic psychological needs. Therefore, the SDT-related interpretations rely on theoretically inferred mechanisms rather than direct need-level evidence. Future research should incorporate validated SDT need-satisfaction measures to more rigorously examine the motivational foundations of digital creativity. Future research should incorporate validated SDT need-satisfaction measures to more rigorously examine the motivational foundations of digital creativity. Second, the quasi-experimental design in Study 1 focused on activating empathic cognitive–emotional states and did not evaluate the objective quality of creativity outputs. Digital creativity was measured through self-reports, which may not fully capture the novelty or usefulness of actual creativity products. Future studies could combine experimental manipulations with performance-based or expert-rated creativity measures. Finally, this study relied primarily on cross-sectional, self-report data, which limits causal inference and the examination of dynamic processes. Future research could adopt longitudinal or experimental designs to explore how digital empathy, cognitive flexibility, and creativity evolve over time and across contexts.

## Data Availability

The original contributions presented in the study are included in the article/Supplementary material, further inquiries can be directed to the corresponding authors.
