# Supplementary material for: From empathy to creative output: exploring the emotional–cognitive mechanisms of digital creativity
Source: Front Psychol. 2026 Feb 25;17:1749596. doi: 10.3389/fpsyg.2026.1749596 (PMC12975487; doi:10.3389/fpsyg.2026.1749596)
Supplement: Supplementary file 1 [file Table_1.DOCX]

Supplementary Material

# Supplementary Data

**Appendix A.** Experimental materials: empathy manipulation.

| Text description | You are a brand packaging designer, currently designing a gift box package for a traditional-culture-themed brand (‘Xiao Guan Tea’). After the brand launched, some consumer comments appeared on social media.  **Consumer A:** The box looks nice, but the overall style feels a bit old-fashioned.  **Consumer B:** It’s stylish, but the price is definitely on the higher side. I’m not sure the packaging justifies the cost.  **Consumer C:** I love the ritual of opening the box. Each tea capsule feels like a small treasure. It’s a beautiful way to present traditional tea culture.  **Consumer D:** The design feels modern but still carries a strong cultural aesthetic. It’s the kind of gift I’d be proud to give to someone.  **Designer’s response:**  Thank you all for sharing your thoughts.  Our goal is to reinterpret traditional tea culture through modern Eastern design. We also hear your suggestions on youthfulness, sustainability, and affordability, and we’ll continue refining our packaging based on your feedback. |
| --- | --- |
| Illustration | 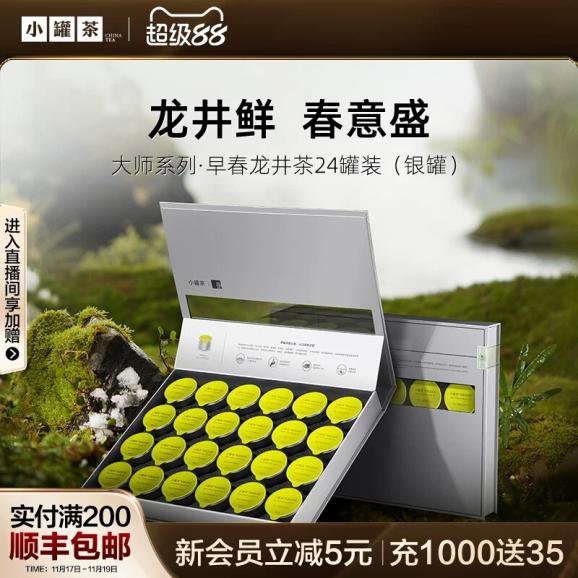 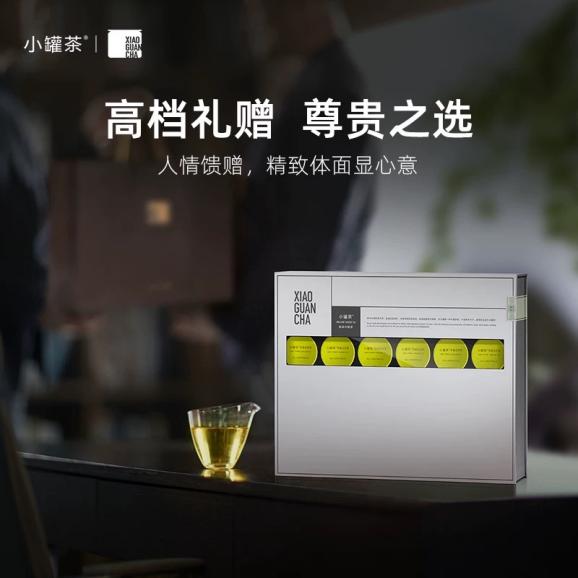 |
| Manipulation | **High-empathy condition**  Please imagine that you are the brand’s packaging designer. As you read the consumer comments, try to put yourself in their position and understand their emotions, aesthetic preferences, and underlying values. Consider what kind of “cultural expression” and “youthful feeling” they hope to see in the packaging. In 3–4 sentences, describe your personal thoughts and feelings at this moment. |
|  | **Low-empathy condition**  Please take the perspective of an external design observer. Describe the consumer comments in an objective and emotionally neutral manner, without taking sides or interpreting their feelings. Identify the main points they raise regarding the packaging design (e.g., traditional tone, youthfulness, cultural aesthetic, pricing). In 3–4 sentences, provide a concise summary of the discussion. |

# Supplementary Data

**Appendix B.**  Normality test result.

| Constructs | N | Mean | S.D. | Skewness | Kurtosis |
| --- | --- | --- | --- | --- | --- |
| DS | 197 | 3.723 | 1.597 | -.031 | -1.496 |
| DE | 197 | 3.808 | 1.695 | .041 | -1.653 |
| DC | 197 | 3.428 | 1.545 | .512 | -.706 |
| CF | 197 | 3.539 | 1.425 | .222 | -1.396 |
| *CF* Cognitive flexibility, *DC* Digital creativity, *DE* Digital empathy, *DS* Digital self-efficacy. | | | | | |

# Supplementary Data

**Appendix C.**  Normality test result.

| Constructs | N | Mean | S.D. | Skewness | Kurtosis |
| --- | --- | --- | --- | --- | --- |
| DS | 303 | 3.758 | 1.651 | -.043 | -1.514 |
| DE | 303 | 3.821 | 1.355 | -.086 | -1.101 |
| DC | 303 | 3.664 | 1.571 | .182 | -1.101 |
| CF | 303 | 3.770 | 1.411 | .066 | -1.382 |
| *CF* Cognitive flexibility, *DC* Digital creativity, *DE* Digital empathy, *DS* Digital self-efficacy. | | | | | |

# Supplementary Data

**Appendix D.** Common method bias assessment using the common method factor (Study1).

| Construct | Indicator | Substantive Factor Loading(R1) | R1^2^ | Method Factor Loading (R2) | R2^2^ |
| --- | --- | --- | --- | --- | --- |
| CF | CF1 | 0.839*** | 0.704 | 0.051 | 0.003 |
|  | CF2 | 0.861*** | 0.741 | -0.216 | 0.047 |
|  | CF3 | 0.835*** | 0.697 | 0.326* | 0.106 |
|  | CF4 | 0.822*** | 0.676 | -0.147 | 0.022 |
|  | CF5 | 0.855*** | 0.731 | -0.008 | 0.000 |
|  | CF6 | 0.840*** | 0.706 | 0.046 | 0.002 |
|  | CF7 | 0.839*** | 0.704 | -0.112 | 0.013 |
|  | CF8 | 0.815*** | 0.664 | 0.191 | 0.036 |
|  | CF9 | 0.828*** | 0.686 | 0.287 | 0.082 |
|  | CF10 | 0.837*** | 0.701 | -0.138 | 0.019 |
|  | CF11 | 0.835*** | 0.697 | -0.148 | 0.022 |
|  | CF12 | 0.787*** | 0.619 | -0.128 | 0.016 |
| DC | DC1 | 0.884*** | 0.781 | 0.048 | 0.002 |
|  | DC2 | 0.877*** | 0.769 | 0.000 | 0.000 |
|  | DC3 | 0.885*** | 0.783 | -0.049 | 0.002 |
| DE | DE1 | 0.909*** | 0.826 | 0.032 | 0.001 |
|  | DE2 | 0.882*** | 0.778 | 0.026 | 0.001 |
|  | DE3 | 0.879*** | 0.773 | 0.140** | 0.020 |
|  | DE4 | 0.871*** | 0.759 | -0.101* | 0.010 |
|  | DE5 | 0.907*** | 0.823 | -0.152*** | 0.023 |
|  | DE6 | 0.843*** | 0.711 | 0.051 | 0.003 |
| DS | DS1 | 0.901*** | 0.812 | 0.016 | 0.000 |
|  | DS2 | 0.864*** | 0.746 | 0.009 | 0.000 |
|  | DS3 | 0.861*** | 0.741 | -0.004 | 0.000 |
|  | DS4 | 0.852*** | 0.726 | 0.061 | 0.004 |
|  | DS5 | 0.871*** | 0.759 | -0.051 | 0.003 |
|  | DS6 | 0.876*** | 0.767 | 0.011 | 0.000 |
|  | DS7 | 0.873*** | 0.762 | -0.041 | 0.002 |
| Average | | 0.858 | 0.737 | 0.000 | 0.016 |
| ** p*<0.05, ***p*<0.01,****p*<0.001, *CF* Cognitive flexibility, *DC* Digital creativity, *DE* Digital empathy, *DS* Digital self-efficacy. | | | | | |

# Supplementary Data

**Appendix E.** Common method bias assessment using the common method factor (Study2).

| Construct | Indicator | Substantive Factor Loading(*R1*) | *R1^2^* | Method Factor Loading (*R2*) | *R2^2^* |
| --- | --- | --- | --- | --- | --- |
| CF | CF1 | 0.840*** | 0.706 | -0.077 | 0.006 |
|  | CF2 | 0.856*** | 0.733 | 0.050 | 0.003 |
|  | CF3 | 0.820*** | 0.672 | 0.321** | 0.103 |
|  | CF4 | 0.809*** | 0.654 | 0.156 | 0.024 |
|  | CF5 | 0.859*** | 0.738 | 0.012 | 0.000 |
|  | CF6 | 0.842*** | 0.709 | -0.262* | 0.069 |
|  | CF7 | 0.819*** | 0.671 | -0.259* | 0.067 |
|  | CF8 | 0.809*** | 0.654 | 0.064 | 0.004 |
|  | CF9 | 0.826*** | 0.682 | 0.114 | 0.013 |
|  | CF10 | 0.833*** | 0.694 | 0.044 | 0.002 |
|  | CF11 | 0.841*** | 0.707 | -0.066 | 0.004 |
|  | CF12 | 0.768*** | 0.590 | -0.096 | 0.009 |
| DC | DC1 | 0.876*** | 0.767 | 0.011 | 0.000 |
|  | DC2 | 0.878*** | 0.771 | -0.009 | 0.000 |
|  | DC3 | 0.880*** | 0.774 | -0.002 | 0.000 |
| DE | DE1 | 0.836*** | 0.699 | 0.066 | 0.004 |
|  | DE2 | 0.851*** | 0.724 | 0.084* | 0.007 |
|  | DE3 | 0.857*** | 0.734 | 0.035 | 0.001 |
|  | DE4 | 0.894*** | 0.799 | -0.09** | 0.008 |
|  | DE5 | 0.856*** | 0.733 | -0.061 | 0.004 |
|  | DE6 | 0.802*** | 0.643 | -0.033 | 0.001 |
| DS | DS1 | 0.890*** | 0.792 | -0.049 | 0.002 |
|  | DS2 | 0.885*** | 0.783 | -0.006 | 0.000 |
|  | DS3 | 0.908*** | 0.824 | 0.024 | 0.001 |
|  | DS4 | 0.905*** | 0.819 | 0.032 | 0.001 |
|  | DS5 | 0.902*** | 0.814 | -0.017 | 0.000 |
|  | DS6 | 0.874*** | 0.764 | 0.035 | 0.001 |
|  | DS7 | 0.879*** | 0.773 | -0.020 | 0.000 |
| Average | | 0.853 | 0.729 | 0.000 | 0.012 |
| ** p*<0.05, ***p*<0.01,****p*<0.001, *CF* Cognitive flexibility, *DC* Digital creativity, *DE* Digital empathy, *DS* Digital self-efficacy. | | | | | |
